# Educator's Blueprint: Optimizing the Resident Research Experience

**DOI:** 10.1002/aet2.70212

**Published:** 2026-06-08

**Authors:** Michael Gottlieb, Richard J. Gawel, Jaime Jordan, Brit Long, Adaira Landry

**Affiliations:** ^1^ Department of Emergency Medicine Rush University Medical Center Chicago Illinois USA; ^2^ Department of Emergency Medicine Hospital of the University of Pennsylvania Philadelphia Pennsylvania USA; ^3^ Department of Emergency Medicine Oregon Health & Science University Portland Oregon USA; ^4^ Department of Emergency Medicine Mayo Clinic College of Medicine Rochester Minnesota USA; ^5^ Department of Emergency Medicine Brigham and Women's Hospital, Harvard Medical School Boston Massachusetts USA

## Abstract

Research is important to advance the field of emergency medicine and improve patient care. As such, emergency medicine residents are required to complete a scholarly project during their training. This experience provides an opportunity to enhance their exposure to research, expanding their skillset as both producers and consumers of the medical literature. However, the process may be overwhelming to launch, especially for residents. This Educator's Blueprint will provide 10 strategies for navigating and optimizing the resident research experience. These strategies include providing structured training, creating a research‐supportive environment, selecting a project using resident‐centric FINER criteria, identifying a research mentor, creating mosaic research experiences, setting expectations, empowering residents, selecting team members for sustainability, providing scaffolding and support during the writing stage, and explaining the peer review process and normalizing rejection or revisions. These strategies will help guide faculty seeking to enhance their learners' research experience.

## 1 Introduction

Research is a core component of academic medicine, helping to advance knowledge in the field and share these findings with others. Early exposure to research can support the development of critical thinking, curiosity, lifelong learning, and professional identity formation while also enhancing the understanding of evidence‐based medicine appraisal during a particularly malleable stage of trainees' careers [1, 2, 3, 4, 5, 6].

Completion of a scholarly activity is also a required component of residency training as defined by the Accreditation Council for Graduate Medical Education (ACGME) [7]. However, studies have shown marked differences in how programs approach the scholarly requirement, prompting some to propose more standardized approaches [8, 9, 10]. One survey study of emergency medicine program directors reported that only 25% of residents presented an oral or poster abstract in the preceding year [8]. Another study reported that 24.3% of American Osteopathic Association residency programs received a citation for not meeting the scholarly activity requirements [11]. A recent cross‐sectional study of eight emergency medicine residency programs using a structured criteria found only half fulfilled all of the requirements for a scholarly project by completion of residency [12]. We acknowledge the broad range of activities that could satisfy the scholarly activity requirement (e.g., book chapters, non‐peer reviewed writing) [7, 10]; however, we believe guidance is particularly warranted for those who chose to work on a research project. While most emergency medicine residents will not pursue a career in research, the skills developed from a research experience can directly translate to a wide array of other arenas [1, 2]. Therefore, there is a need for a consolidated, practical, and adaptable set of strategies that programs can implement regardless of size, resources, or existing infrastructure.

The purpose of this manuscript is to synthesize the current evidence along with the authorship groups' combined experience mentoring hundreds of students, residents, and junior faculty to provide 10 practical, actionable strategies for programs to optimize the resident research experience in a manner that is adaptable across diverse program structures. The authorship group represents emergency medicine residency programs of varying size, geographic location, and research infrastructure, including both faculty and a current resident, with collective experience in program leadership, faculty development, research mentorship, and medical education scholarship. The authorship group has collectively authored and mentored learners on more than 1000 peer‐reviewed publications. The goal of this Educator's Blueprint is to provide educators and program leadership with a blueprint that improves research productivity, mentorship quality, educational outcomes, resident satisfaction, and long‐term academic engagement. By outlining a set of practical strategies grounded in best practices, this manuscript seeks to strengthen how residency programs conceptualize, structure, and support resident research activities.

## 2 Ten Strategies

### 2.1 Provide Structured Training

Many emergency medicine residents enter training with variable interest in and previous exposure to research [13, 14]. Acknowledging this gap in preparatory training, residency programs should provide a structured research curriculum to ensure foundational skill development. It has been recommended that residency research training should be longitudinal, spanning all years of residency and gradually increasing in complexity and responsibility [15]. Prior work has demonstrated that structured, multi‐year curricula improve resident productivity, increase research output, and enhance understanding of core research concepts [16, 17, 18]. Longitudinal structure also creates predictable expectations and allows residents to develop skills iteratively by sequentially building upon existing knowledge.

While the specific design will vary by program, residency leadership should consider adopting a multimodal approach that incorporates live didactic sessions during residency education conferences, individualized mentorship meetings, and asynchronous learning tools. Asynchronous resources, such as modular online content, curated reading lists, and self‐paced activities, allow residents to engage with core principles on their own schedule and reinforce concepts introduced during didactics or mentorship meetings. Competency‐based microlearning platforms, including digital badge programs, have also shown promise in promoting self‐directed skill acquisition and improving learner confidence [19]. More recently, there have been proposals to engage artificial intelligence for providing structured feedback and practice datasets with an emphasis on formative growth [20].

In addition, programs may benefit from offering dedicated research elective experiences to provide focused time for deeper engagement with ongoing projects. These electives can facilitate regular mentor meetings, advance data collection or analysis, and provide dedicated time for manuscript writing, particularly in four‐year programs with greater scheduling flexibility [21]. When paired with a structured curriculum and accessible asynchronous tools, dedicated electives create a coherent and structured learning program that fosters consistent research skill development throughout residency.

**FIGURE 1:**
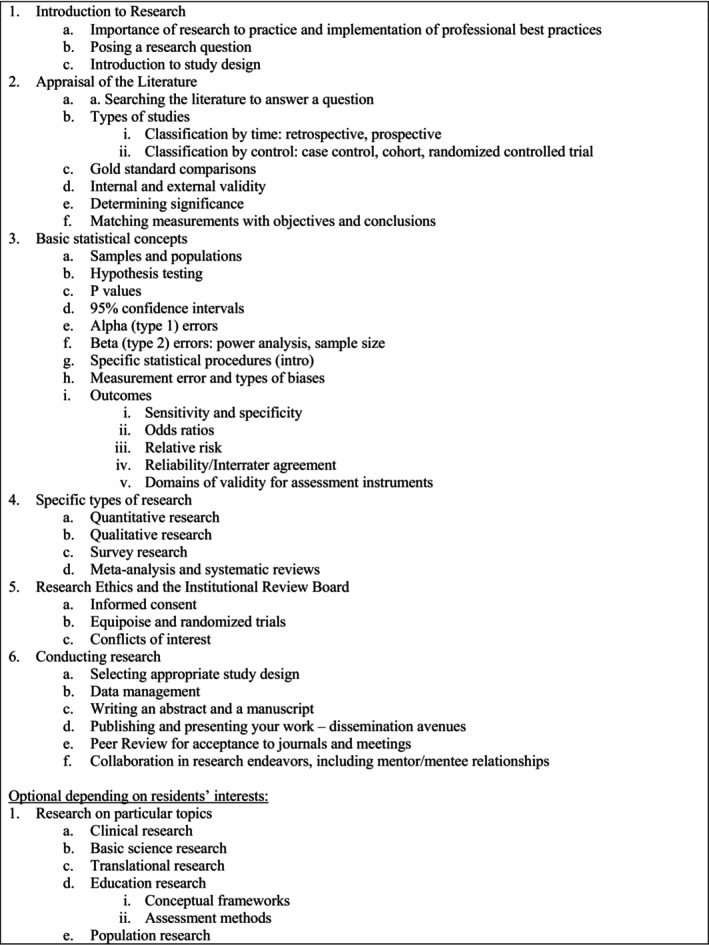
Sample model research curriculum from Hartman et al. [15].

Barriers to implementation include limited faculty capacity to develop and deliver longitudinal curricula; competing demands on conference time; and absence of local content experts in research methodology, biostatistics, or scholarly writing. Smaller or community‐based programs may not have a dedicated research director or sufficient research‐active faculty to staff a multi‐year curriculum. Programs facing these constraints can use publicly available curricula and online resources to supplement local efforts. Examples include offerings from the Society for Academic Emergency Medicine (SAEM) and SAEM Academies, the Council of Residency Directors in Emergency Medicine (CORD) educational resources, MedEdPORTAL teaching materials, and free online courses through platforms such as Coursera and edX. Programs can also consider partnering with neighboring academic institutions or specialty societies to share didactic content, faculty expertise, or research workshops. Suggested topics for a longitudinal resident research curriculum are outlined in Figure 1 [15].

### 2.2 Create a Research‐Supportive Environment

A robust research experience depends not only on structured curricula, but also on the broader environment in which residents learn [22]. One foundational element is transparency around available mentors, their areas of expertise, and their willingness to involve residents in their work. Programs should maintain an easily accessible listing of research‐active faculty that includes brief descriptions of their interests, ongoing projects, and recent publications. This helps residents, especially those unfamiliar with the academic landscape, understand the range of opportunities within their department and identify potential mentors whose work aligns with their interests. Centralizing this information on an internal website or dedicated research page ensures that it remains visible, up‐to‐date, and usable.

Equally important is providing clarity around the local research infrastructure and how residents can access it. Departments should explicitly outline available institutional resources, such as medical librarians, statisticians, the institutional review board (IRB), and funding opportunities and provide guidance on how to engage each service. Organization of resources may reduce barriers that commonly impede novice investigators and reinforce that research is an institutional priority rather than an individual burden.

Finally, residency programs can strengthen a research‐supportive environment by purposefully incorporating scholarly development into the academic calendar [23]. Building protected time into conference schedules (e.g., periodic research workshops or focused discussions led by research faculty) creates consistent exposure to fundamental concepts such as critical appraisal, study design, and data interpretation. Having faculty present their own work in a more holistic manner (i.e., including not only study outcomes but also an overview of the mechanics of and lessons learned from creating the project) during these sessions enhances relevance, demystifies the research process, and naturally creates entry points for residents to join ongoing projects. Programs should also create structured opportunities for residents to present their work to peers and faculty, such as departmental research days or scholarly symposia. These venues, especially if paired with a question and answer section, promote confidence and accountability, enhance communication skills, and expose residents to the breadth of ongoing research activity within their department. Collectively, these structured touchpoints can cultivate an environment in which research feels integrated into emergency medicine training rather than an optional extracurricular activity, ultimately increasing engagement across the residency program.

Barriers to implementation include a limited number of research‐active faculty available to populate a mentor list, lack of institutional infrastructure such as access to dedicated biostatisticians or research librarians, and competing curricular priorities that constrain protected time on the academic calendar. In community‐based or smaller programs, identifying even a small core of research‐active faculty can be challenging [11]. To address these barriers, programs can partner with affiliated academic centers, hospital research offices, or university libraries to extend resources, designate a single research liaison faculty member to maintain the mentor directory and connect residents to external resources, and use virtual research collaboratives or national networks (e.g., SAEM Academies, Emergency Medicine Residents Association [EMRA], CORD Academy for Scholarship) to expand access. Programs without departmental research days can co‐host scholarly symposia with neighboring institutions or use existing grand rounds slots to feature trainee work. Programs should also visibly celebrate resident scholarly accomplishments through newsletters, awards, conference recognition, or departmental communications to reinforce the value of academic engagement.

### 2.3 Select a Project Using Resident‐Centric FINER Criteria

Faculty play a key role in advising and guiding residents on project selection. Appropriate selection of a research project is a critical step to navigating collaboration, skill acquisition, reputation building, and academic publication. Understanding and participating in the process of selecting a research project is an instrumental experience for all residents, even those who do not wish to have a career in academic research [24]. The FINER (feasible, interesting, novel, ethical, relevant) criteria can be particularly valuable for resident researchers to serve as a practical situational assessment of the appropriateness of a given project idea [25].

When assessing for “feasibility,” residents should consider the scheduling limitations of residency, realistic access to data and funding, the availability and reliability of collaborators, and the need for technical support, such as learning how to capture and analyze data. This is a mindset shift from “Should this study be done?” to “Can this study be done?.” A study of internal medicine residents found that only 55% of projects made it to completion, as evidenced by publication [26]. Similar trends have been found in other fields, including emergency medicine [8, 27, 28, 29]. Feasibility also requires mapping the project onto a concrete training timeline asking whether key steps (e.g., IRB approval, data collection, analysis and manuscript preparation) can be completed within the residency window and determining what existing infrastructure (e.g., registries, prior datasets, manuscript templates, research staff) can be leveraged instead of building the entirety of the project de novo.

When considering if a project is “interesting” or “novel,” residents should reframe aspirations away from conducting groundbreaking and paradigm‐shifting research and shift toward finding a project that feels sustainably engaging and also locally meaningful. Rather than studying a question that is complex or fringe, novice resident researchers may be better served by choosing straightforward topics that align with their clinical or educational interests first. After picking a topic of interest, residents should consider designing a study by replicating or adapting prior work that has been done in their local setting or published in a relevant journal. This might translate to studying a known problem in a new population, implementing an established intervention at a different institution, or focusing on a narrow but important piece of a larger question that has been previously studied. Approaching novelty in this incremental way lowers the threshold to start research while still meaningfully contributing to the literature or local practice.

Evaluation of whether a study is “ethical” should also be considered within the unique resident research experience. Ethical considerations include not only participant protections in the research itself but also avoiding designs that are so complex or resource intensive that they are at high risk of protocol deviations, missing data, or having enrollees that are lost to follow‐up. Faculty and trainees should also consider the hidden harms with projects that have a low likelihood of successful completion (e.g., resident time and motivation, participant time, emergency department throughput), and ensure projects are selected with a high likelihood of completion.

“Relevance” can be reframed to problems that matter in the resident's immediate environment (e.g., recurrent gaps in patient care, workflow efficiencies, curricular needs, equity issues). By leveraging local issues as a foundation, the project's findings are better positioned to be translated into visible local changes, thereby increasing motivation and engagement. An academic guide providing a more in‐depth review of project selection for residents has been published previously [30].

Barriers to implementation include resident inexperience in critically assessing feasibility, faculty who may not be familiar with the FINER framework, and pressure to pursue ambitious projects that exceed available resources or time. Faculty may also feel uncertain about how to redirect a resident away from a project that is not appropriate without dampening enthusiasm. To address these barriers, programs can incorporate brief teaching on the FINER criteria during early research curriculum sessions; provide faculty development on guiding project selection conversations; and offer a curated list of feasible local data sources, registries, or smaller‐scale project templates that residents can build upon. Programs may also consider implementing a structured project proposal review process in which a faculty committee provides early feedback on feasibility and scope before substantial work begins.

### 2.4 Identify a Research Mentor

Selecting the right research mentor(s) can be one of the most influential determinants of a resident's scholarly success [1]. In the context of residency, mentors should view themselves not simply as supervisors of research tasks but as facilitators of professional identity formation [5, 6]. Emphasizing this broader framing to potential research mentors can help to ensure they better understand their potential impact, which can extend beyond the mechanics of simply producing a research project to shape how residents view themselves within academic medicine, how they develop confidence and scholarly curiosity, and whether they continue to engage in scholarship beyond training [1]. The overarching goal is therefore not merely to complete a project, but to build the skill set and confidence that can guide, empower, and sustain scholarly engagement throughout a mentee's career.

Contemporary mentorship frameworks provide useful guidance on how research mentors can achieve this goal. Most and Sikora describe five research advisor maxims: professional generosity, maximizing “bang for buck,” servant leadership, finishing the drill, and truthfulness [31]. These five pillars align with the five ethical pillars of research mentorship: nonmaleficence, autonomy, fidelity, beneficence, and honesty [32]. This framework emphasizes both the relational and operational components of effective mentorship. Professional generosity and beneficence encourage mentors to share opportunities and provide psychological safety. Examples of this include sponsoring or supporting residents to attend conferences, take courses, or apply for grants and awards. It also involves the mentor providing constructive feedback in a compassionate and graceful manner. Autonomy and maximizing “bang for buck” support the development of resident ownership and scholarly independence. While it may be easier to complete a task as a mentor, the true test of allowing space to grow is providing time and opportunity for a mentee to iterate and learn from their choices. Fidelity and finishing the drill reinforce the expectation of project completion and follow‐through. Mentors should, with grace and compassion, still hold residents accountable for work that has stalled and remained incomplete. Finally, honesty and truthfulness promote shared understanding, realistic timelines, and transparency about barriers. These maxims are relevant for all stages and fields of practice in academic medicine and suggest pragmatic language for describing the responsibilities of research mentors. In the context of residents, however, these factors should be heavily considered when recommending or agreeing to serve as a potential mentor.

Programs themselves also play a critical role in helping residents identify and connect with effective mentors. Relying on informal matchmaking or pairing strategies, such as opportunistic hallway introductions, self‐initiated cold outreach, or even warm introductions by mutual contacts, may be successful though more often than not can lead to inadequate access and inconsistent outcomes [33, 34]. More intentional approaches by the program include: (1) creating formal residency mentorship programs that assign or pair residents with research‐active faculty; (2) providing residents with accurate lists of potential mentors that include areas of expertise, methodological experience, schedule availability, and recent projects; and (3) delaying required mentor selection until residents have acclimated to the clinical environment and gained clarity around their interests and workflow [22, 35, 36, 37]. Allowing a brief period of adjustment after arriving into the residency program (i.e., typically the first several months of the first year) can improve alignment between mentor interests and resident goals and may prevent early mispairings that require remediation in later months or years [33].

Research mentorship opportunities may vary across institutions. In settings where local research mentorship capacity is limited, programs should consider expanding the landscape of mentorship to include inter‐institutional arrangements, peer and near‐peer mentorship, and interdepartmental or interdisciplinary arrangements [38, 39, 40]. External mentorship can be facilitated through existing academic relationships, national organizations, virtual research collaboratives, or specialty societies [41, 42]. Peer and near‐peer mentorship models, such as senior residents supporting junior residents, can increase psychological safety, empower senior residents to take on more of a leadership and mentoring role, and enhance continuity as projects progress over time. These peer and near‐peer relationships may provide a more comfortable environment for residents to ask their peers about working with particular faculty mentors. However, it is also important to include more experienced mentors, as data suggest more experienced mentors can lead to increased project completion and eventual publication [28].

Mentorship also needs to consider equity and ensure that all residents feel supported. Residents from historically underrepresented groups may face additional barriers in accessing mentorship networks, identifying aligned role models, or navigating academic spaces [43, 44]. One study of medical students found that women and those underrepresented in medicine (URiM) publish less than white men, with URiM women publishing the least of all cohorts [45]. Programs should therefore consider intentional strategies to support residents who are URiM, such as highlighting affinity‐based mentorship platforms, connecting residents with national mentorship networks (e.g., FeminEM, EMRA), and ensuring visibility of diverse mentors within the local environment. Additional concrete steps include establishing formal sponsorship programs in which senior faculty actively advocate for URiM residents by nominating them for awards, speaking opportunities, and authorship positions; providing dedicated funding to support attendance at affinity‐group conferences or leadership development programs; pairing URiM residents with multiple mentors who can address distinct domains (e.g., research, career navigation, identity‐based support) rather than relying on a single mentor to address all needs; and creating structured check‐ins between program leadership and URiM residents to identify and address barriers proactively [46, 47]. Programs should also examine internal data on resident research productivity by demographic group to identify and address disparities and ensure that faculty mentors receive training on inclusive mentorship practices.

Barriers to implementation include a shortage of available faculty mentors, lack of methodological diversity among existing mentors, and time constraints that limit faculty capacity to take on new mentees. Faculty who lack formal mentorship training may also feel underprepared to guide residents through the research process. To address these barriers, programs can develop faculty mentorship training opportunities, formally recognize mentorship in promotion and compensation structures, and build mentorship networks that extend beyond the home institution through inter‐institutional and virtual arrangements. When local capacity is truly limited, peer and near‐peer mentorship can supplement faculty oversight, with faculty providing strategic guidance at key project milestones rather than continuous direct supervision.

### 2.5 Consider Mosaic Research Experiences

While it is optimal to select a resident research project that can be completed during the length of their residency training, that is not always possible due to both project‐related and external factors (e.g., COVID‐19, shift in patient population). Traditionally, the research requirement has been considered in the context of a singular project, which can potentially lead to either incomplete projects or overly simple projects with minimal research focus. To address this need, some have proposed expanding the definition or approach to increase the ability to successfully complete a scholarly project. Modeled after scholarly frameworks from Boyer and Glassick [48, 49, 50], Pillow and colleagues proposed a novel approach by using multiple separate projects to meet the key elements of the scholarly requirement when a single project does not meet that need (Table 1) [10]. This model can also facilitate clear reporting to meet ACGME guidelines, while also ensuring that the resident can complete each of the key elements of the scholarly process.

**TABLE 1:** Rubric for residents engaging in scholarly projects across multiple projects.

Domains	Key components	Checklist for each item
Process	Clear goals	
	Adequate preparation	
	Appropriate methods	
Outcomes	Verifiable Results	
Dissemination	Effective presentation	
Peer review and feedback	Reflective critique	

*Source:* Adapted from Pillow et al. [10]^.^

Another model that one of the authors of this blueprint is the Time‐Independent Project Ownership for Fellows (TIPOFF) model, wherein a resident or fellow who wishes to join an existing project is challenged to first design the project de novo, then undergoes mentored refinement, and finally integrates with the team [51]. The advantage of this latter model is that the resident still learns the project development and refinement stages before joining the study, thus gaining more ownership and confidence in project creation despite not joining at the onset.

Barriers to implementation include the additional administrative burden of tracking scholarly progress across multiple smaller projects rather than a single longitudinal effort, faculty unfamiliarity with the mosaic framework, and the perception that fragmented contributions are less rigorous than a traditional single‐project experience. Communicating these models to ACGME reviewers may also require additional documentation. To address these barriers, programs can adopt the rubric in Table 1 as a structured reporting tool, train faculty mentors on the mosaic approach during faculty development sessions, and ensure that residents understand from the outset that the goal is meaningful engagement with the full scholarly process rather than completion of a singular product. Programs should also clearly document the specific component each project contributes to the overall scholarly experience.

### 2.6 Set Expectations

Expectations should be set at both the program and the project level so that residents understand what is required of them from the beginning of training. Program‐level expectations should include the scope of the scholarly requirement, the timeline for project identification and completion, the resources available, and how progress will be evaluated. Communicating these expectations early, through orientation materials, the resident handbook, and structured meetings with program leadership, allows residents to plan accordingly and engage meaningfully with each step. The rubric outlined in Table 1, or an alternative tool, can be used to make program‐level expectations transparent and explicit.

Prior to initiating the project, the research mentor should be sure to establish project‐level expectations for roles, communication, and timelines with full transparency. This discussion can be framed under the promise that an upfront discussion of expectations will help ensure scholarly project completion. Residents who have not worked on an academic project prior to residency may have limited knowledge of and experience with the different roles of an academic project. By discussing author position and corresponding expectations early, all members of the research team can ensure that they meet the requirements of their role and any ill feelings about discordant effort are reduced at later stages [52]. This should also include a discussion of the authorship criteria. The International Committee of Medical Journal Editors (ICMJE) recommends authorship be based on four criteria, including: (1) substantially contributing to the conception or design of the work; or the acquisition, analysis, or interpretation of data for the work; and (2) drafting the work or reviewing it critically for important intellectual content; and (3) approving the final version to be published; and (4) agreeing to be accountable for all aspects of the work in ensuring that questions related to the accuracy or integrity of any part of the work are appropriately investigated and resolved [53]. In addition to general author roles, residents should also clarify who should be the primary contact for communication with the authorship team. Similar to a resuscitation in the clinical setting, it can be beneficial for the resident to experience the role of team leader with appropriate supervision based on their experience and skills. This requires balancing autonomy and support. If the mentor is too hands‐on, the resident will not develop the critical skills in project management; in contrast, if the mentor is too hands‐off, the project may not progress or the resident may direct the project in the wrong direction.

The mentor and mentee should also establish a timeline and approach to communication [1]. The timeline should be established early, preferably during the initial meeting with the resident about the project [31, 36, 39, 54]. The timeline should be broken down into specific stages (e.g., literature review, study design, IRB submission) with discrete deadlines for each element. The mentor should also advise the resident to factor in rotations that are higher volume or have greater time commitments as well as vacation time. To allay the risk of burnout or resentment, residents should not be expected to spend their vacation working on a research project. While individual residents may choose to do so, this should not be a faculty expectation. Proper and realistic planning can help schedule meetings and deadlines so that professional work does not critically invade personal time. Project timelines should also include regularly scheduled meetings with the resident and research team members, which will help ensure the project remains on track. As unexpected barriers will most certainly arise, time should be added to the deadline to account for any unforeseen situations or issues.

Barriers to implementation include faculty discomfort with explicit conversations about authorship and expectations, varying program‐level standards for what constitutes adequate scholarly work, and difficulty enforcing timelines when residents face unexpected clinical or personal demands. To address these barriers, programs can provide template expectation‐setting documents or contracts that mentors and residents complete together at project initiation, offer faculty development sessions on how to lead these conversations, and disseminate authorship criteria (e.g., ICMJE) widely so that they become routine reference points. Program leadership can also normalize timeline revisions as part of the research process rather than treating them as failures.

### 2.7 Empower Residents

Empowerment serves as a foundation for professional identity formation, the process through which residents integrate the roles, values, and behaviors of their profession into their self‐concept [55]. When residents take ownership of scholarly work, they begin to see themselves as contributors to the academic mission of medicine rather than merely consumers of knowledge. Conducting scholarship has been noted to aid trainees in focusing their professional interests and informing their career decisions, helping them find their niche, clarify career goals, and envision diverse professional pathways, including but not limited to academic medicine [1, 2]. This grounding rationale informs why empowerment, rather than mere task assignment, is a critical component of the resident research experience.

Optimizing the resident research experience requires intentionally empowering residents as owners of their scholarly work, rather than positioning them as passive contributors to faculty‐driven projects. Empowerment in this context means that residents perceive the project as their project—even when closely mentored—thereby fostering intrinsic motivation, accountability, and persistence. This mirrors the clinical training model in which junior residents assume primary responsibility for their patient under supervision, a structure known to promote engagement, learning, and professional growth and eventually lead to mastery [56].

This approach is strongly supported by Self‐Determination Theory, which posits that motivation and performance are optimized when learners experience autonomy, competence, and relatedness [57, 58, 59]. Supporting autonomy in resident research involves allowing residents to meaningfully shape the research question, methods, and dissemination strategy, rather than limiting their role to executing predefined tasks. Evidence from graduate medical education literature reinforces the importance of resident ownership in scholarly activity. Residents are more successful when they perceive scholarly work as personally meaningful and when their autonomy is promoted [1, 60]. Promoting resident autonomy deepens their involvement in the experience, increasing its meaning and value [1].

Importantly, empowerment does not imply absence of structure. Too much autonomy or a lack of supervision can be detrimental to trainee success [1]. Particularly for early learners, faculty mentorship should include intentional review of residents' plans for project completion, with attention to feasibility, timelines, methodological rigor, and anticipated barriers. Early identification of gaps supports competence development while preserving autonomy [56]. Providing responsive mentorship while still allowing residents' ideas and vision to guide the project is important for successful completion of scholarly work [1].

Barriers to implementation include faculty members' reluctance to relinquish control over their own research agendas, time pressures that make it more expedient to assign discrete tasks rather than support independent project development, and inexperienced residents who may not yet recognize their own ideas as valuable contributions. Some residents may also lack confidence in proposing or modifying study designs. To address these barriers, programs can train faculty mentors on graduated autonomy approaches, encourage mentors to allocate dedicated time for collaborative brainstorming early in the project, and create low‐stakes opportunities for residents to practice generating and defending research ideas (e.g., research idea pitches at journal club or research conferences). Recognizing and celebrating resident‐led contributions in departmental communications can also reinforce the message that resident ownership is valued.

### 2.8 Select Team Members for Sustainability

Sustaining resident research in an optimal fashion requires intentional team composition that anticipates resident turnover and the longitudinal nature of many research projects. A practical and effective strategy is the use of staggered resident involvement, such that at any given time each project includes a senior resident, a junior resident, and a faculty mentor. This structure promotes continuity, distributes responsibility across training levels, and supports sustained progress over multiple academic years.

Within this model, the senior resident functions as a near‐peer mentor, providing continuity as projects advance from data collection to analysis, manuscript preparation, and dissemination. Near‐peer teaching and mentorship have many benefits including enhanced learning efficiency, reinforcement of knowledge and teaching skills for senior trainees, increased professional satisfaction, and normalization of challenges for junior learners [61, 62, 63, 64, 65]. The junior resident gains early exposure to the full research lifecycle, builds foundational skills, and progressively assumes ownership, positioning them to become the senior steward of the project in subsequent cycles. This intentional overlap facilitates knowledge transfer and mitigates loss of momentum when residents graduate.

Team‐based structures can also allow scholarly work to persist despite competing clinical demands of residents. By strategically composing teams of different levels of residents, with varied clinical schedule blocks, faculty mentors can mitigate a common barrier to completing scholarship for residents: lack of time [1]. This type of structure can allow one team member to assume a more active role during times of greater capacity, while others step in when they have more capacity, ensuring that the project does not stagnate. Additionally, social cognitive career theory suggests that observing near‐peers successfully navigate scholarly roles enhances self‐efficacy and sustains engagement in work [66, 67]. The self‐efficacy of senior residents on the team can also be enhanced by having junior residents seek out their advice [67].

Faculty mentors play a critical anchoring role, maintaining longitudinal oversight while enabling residents to lead discrete phases of the work. By deliberately selecting team members with staggered roles, residency programs can create durable scholarly teams with shared responsibility that maintain momentum, foster self‐efficacy, and support learner development across stages of training, increasing the likelihood of meaningful scholarly output.

Barriers to implementation include limited numbers of residents interested in a similar topic within a single program to staff multiple staggered teams, mismatched schedules that make handoff meetings difficult, and inconsistent commitment levels across team members that can slow progress. Faculty mentors may also find it challenging to coordinate multiple residents at different training levels on a single project. To address these barriers, smaller programs can adopt cross‐class teams composed of as few as two residents (one senior, one junior) and a faculty mentor, recruit residents from affiliated programs to participate as collaborators, and establish brief structured handoff documents that capture project status, immediate next steps, and outstanding questions when a team member departs. Programs can also schedule recurring asynchronous check‐ins as a group or one‐on‐one meetings with team members to ensure continuity when synchronous meetings are not feasible [68, 69].

### 2.9 Provide Scaffolding and Support During the Writing Stage

The majority of emergency medicine residents have not written a scientific manuscript prior to residency [70]. Therefore, it is important to provide a structure for writing each section. This can be accomplished through one‐on‐one discussions, asynchronous resources (including journal websites and published reporting guidelines), and review of previously accepted articles. In doing so, it is important to help guide them through the key elements that should be included in each section, the traditional order of reporting, and avoidance of common errors (e.g., reporting results in the methods section, presenting new data in the discussion section). Providing a scaffold outlining the sections and subsections can make it easier to determine where items go within each portion of the paper. In addition, junior authors will benefit from more direct support at the onset. One technique the authors have previously used is writing dyads, where each section has a junior and senior author, such that the junior author has a clear designated mentor that can allow for more rapid feedback and a lower barrier to reaching out as opposed to querying the entire authorship team. With each paper, the junior author rotates across one or more sections, gaining in‐depth experience with a new aspect each time. By allocating the dyad writing mentors to fewer sections, the mentor is encouraged to invest more time in the edits and feedback. For example, rather than rewriting a section of the paper, the mentor would add comments and provide specific examples of how the writing could be improved. Notably, since less‐experienced writers may not be familiar with the editing and feedback process, it is also important to emphasize to learners at the onset that edits are a common and expected process in academic writing and should be seen as a sign of investment by the mentor. It is important for residents to know that if they receive significant edits by a team member that this is not a marker against their self‐worth or promise. A mentor or peer who spends significant time editing a manuscript is demonstrating their investment in the resident and project. Remind the resident it is a normal process to get heavy edits as one develops their scientific writing skills.

Barriers to implementation include limited faculty time to provide detailed editorial feedback, faculty who themselves may not feel confident in their scientific writing skills, and resident reluctance to share early drafts for fear of judgment. To address these barriers, programs can use existing reporting guidelines (e.g., STROBE, CONSORT) and published writing guides to standardize feedback and reduce the cognitive load on individual faculty. Programs can also encourage the use of writing groups in which residents and faculty review each other's work, which distributes the editorial burden and creates a culture of shared learning. Faculty development opportunities in scientific writing, available through local and national programs, can build mentor capacity [71, 72, 73, 74, 75, 76]. Normalizing the use of drafts, including imperfect ones, in mentorship meetings helps residents view writing as iterative rather than evaluative.

### 2.10 Explain the Peer Review Process and Normalize Rejection or Revisions

Prior to submitting a manuscript, it is valuable to discuss the peer review process and the accompanying timeline [77, 78]. During this stage, it can be beneficial to set expectations regarding acceptance rates and to normalize that rejection is common, with one study reporting that the mean acceptance rate for original research in emergency medicine is 25% [79]. One approach to address this early on is to identify multiple target journals so that the manuscript can easily be moved to the next journal if declined, thereby reducing the negative feelings associated with rejection and maintaining momentum toward publication. As many residents may not have experience with selecting appropriate journals, faculty should help guide them through appropriately tailored journals. Strategies to improve journal selection include reviewing prior publications that the manuscript has cited, using online resources (https://jane.biosemantics.org/, https://journalfinder.elsevier.com/), and querying mentors and peers. One important element is to avoid predatory journals that have limited impact and profit from inexperienced authors by charging exorbitant publication fees without providing the typical commensurate publishing services [52, 80]. In addition to submitting manuscripts, discussing the importance of abstract presentations at conferences is helpful. Conference presentations can serve as a checkpoint and early win for ongoing projects (particularly if the data analyses will not be completed until after the resident graduates), foster early exposure to present research in a national setting and develop their public speaking skills, and provide an early, unique opportunity to network with others in the field [41, 81, 82].

Barriers to implementation include resident discouragement following rejection, faculty mentors who are themselves unfamiliar with the breadth of journal options or the nuances of journal selection, financial constraints related to open‐access publication fees, and limited departmental funding to support conference travel. To address these barriers, programs can build brief sessions on the peer review process and journal selection into the research curriculum, encourage faculty to share their own rejection and revision experiences openly to normalize the process, and provide guidance on identifying journals with no or reduced fees. Programs should also explore institutional funding for resident conference travel and consider supporting attendance through grants, departmental funds, or specialty society awards. Pairing each manuscript submission with a structured debrief, regardless of outcome, can help residents extract learning from the experience and maintain motivation.

## 3 Conclusion

In this article, we described 10 strategies for optimizing the resident research experience. Educators can utilize these strategies when designing resident research experiences.

## Author Contributions

**Michael Gottlieb:** conceptualization, writing – original draft, writing – review and editing, supervision. **Richard J. Gawel:** conceptualization, writing – original draft, writing – review and editing. **Jaime Jordan:** conceptualization, writing – original draft, writing – review and editing. **Brit Long:** conceptualization, writing – original draft, writing – review and editing. **Adaira Landry:** conceptualization, writing – original draft, writing – review and editing.

## Funding

The authors have nothing to report.

## Conflicts of Interest

The authors declare no conflicts of interest.

## Data Availability

Data sharing not applicable to this article as no datasets were generated or analyzed during the current study.
